# Rapid Surgeon-Led 3D Printing of Artificial Intelligence-Assisted Cylindrical Templates for Visceral Vessel Fenestration in Physician-Modified Endografts

**DOI:** 10.7759/cureus.97860

**Published:** 2025-11-26

**Authors:** Juan Carlos Gomez-Rodriguez, Jesus Rosso, Barbara Dieck, Edison Peña, Andres Cadavid

**Affiliations:** 1 Vascular Surgery Department, Clinica Medellin, Medellin, COL; 2 Vascular Surgery Residency Program, Universidad de Antioquia, Medellin, COL

**Keywords:** ‎3d printing, aortic abdominal aneurysm, artificial intelligence in healthcare, endovascular aortic repair, fenestrated endovascular aneurysm repair, physician-modified endografts

## Abstract

Endovascular aortic aneurysm repair (EVAR) of complex abdominal aortic aneurysms (AAAs) requires precise preoperative planning to ensure proper alignment of visceral fenestrations. However, commercial planning platforms remain costly and often inaccessible for many vascular centers. Low-cost, surgeon-driven digital workflows utilizing open-source software and desktop three-dimensional (3D) printing have become practical alternatives, allowing patient-specific planning without dependence on proprietary systems. In this report, we describe a reproducible digital workflow that combines open-source imaging software for centerline extraction, anatomical segmentation, and stereolithography (STL) model creation, along with artificial intelligence (AI)-assisted scripting to generate cylindrical fenestrated templates. The anatomical and cylindrical models were processed and printed on an affordable desktop fused-deposition modeling (FDM) printer using polylactic acid (PLA) filament. The anatomical reconstruction provided spatial orientation, while the cylindrical template served as an accurate guide for physician-modified endografts (PMEGs). A 78-year-old man with an infrarenal AAA and a prior failed EVAR underwent successful reintervention using this workflow. Fenestrations for the celiac trunk, superior mesenteric artery, and renal arteries were planned along the centerline, validated against the anatomical model, and accurately transferred to the endograft using the cylindrical stencil. Final angiography confirmed aneurysm exclusion and visceral branch patency. The cylindrical template took less than two hours to print and was sterilized with low-temperature plasma before intraoperative use. This case demonstrates that a fully open-source digital workflow, integrating centerline analysis, anatomical segmentation, STL generation, and AI-assisted modeling, can produce precise and reproducible intraoperative guides for PMEG planning. The process is quick, low-cost, and scalable, providing a viable alternative for centers lacking access to commercial planning software or custom-made devices.

## Introduction

Endovascular aortic aneurysm repair (EVAR) has become the standard of care for infrarenal abdominal aortic aneurysms (AAAs), providing reduced perioperative morbidity compared to open repair. However, complex anatomies such as juxtarenal or pararenal AAAs remain technically demanding due to inadequate proximal neck length. In this context, fenestrated and branched endovascular aneurysm repair (FEVAR/BEVAR) techniques have emerged as safe and effective options, with high technical success rates and low early mortality, even in high-risk or elderly populations [1-3]. Despite these advancements, the success of fenestrated repair depends greatly on meticulous preoperative planning, precise device customization, and careful intraoperative execution.

Over the last decade, three-dimensional (3D) printing has revolutionized surgical planning by providing patient-specific aortic models that enhance anatomical understanding, simulation, and training [4-8]. In vascular surgery, printed replicas have been used for preoperative rehearsal, device sizing, and education, leading to reductions in operative time and contrast use [9]. Beyond visualization, 3D-printed templates have guided physician-modified endografts (PMEGs), achieving high alignment accuracy for renal and visceral fenestrations [10]. These methods emphasize the clinical importance of incorporating digital modeling into the workflow of complex EVAR.

Open-source software has played a key role in democratizing these innovations. 3D Slicer (Brigham and Women’s Hospital and Harvard Medical School, Boston, MA, USA), widely used in vascular image segmentation, enables surgeons to create precise 3D reconstructions of the aorta and its branches from computed tomography angiography (CTA) data [11,12]. When combined with the Horos (Horos Project, Purview, Toronto, Canada) Vascular Modeling Toolkit (VMTK), it offers advanced tools for centerline extraction, vessel analysis, and quantitative planning. Likewise, Blender (Blender Foundation, Amsterdam, Netherlands), a 3D graphics platform with Python scripting capabilities, has become increasingly valuable in medical modeling, enabling improvements to anatomical meshes, the addition of wall thickness, and the development of fenestration guides [13]. By utilizing Blender’s scripting application programming interface (API), surgeons can design automated workflows for patient-specific device planning, overcoming the limitations of costly proprietary software.

Simultaneously, artificial intelligence (AI) is transforming vascular surgery through automated image analysis and predictive modeling. Recent research shows that AI algorithms can segment CT scans with nearly expert-level accuracy, automate aortic measurements, and even predict outcomes after open or endovascular repair [14-16]. These technologies have the potential to reduce inter-operator variability, expedite case preparation, and support personalized risk assessment, especially in complex aortic diseases.

Importantly, several groups from Latin America have contributed to disseminating low-cost, surgeon-led digital workflows for EVAR planning. In 2022, our group described a cost-effective toolkit for AAA sizing and 3D printing, achieving acceptable accuracy at a fraction of the cost of commercial solutions [17]. In 2024, we expanded this concept in an IntechOpen book chapter, presenting a comprehensive pipeline that combines Horos, 3D Slicer, Blender, and vector-based design software to generate fenestration templates for PMEGs [18]. More recently, augmented reality and intraoperative image fusion have been integrated into this framework, further demonstrating the versatility of low-cost digital tools in complex EVAR [19].

Overall, the literature supports a growing paradigm shift: surgeons can directly lead the integration of 3D printing, open-source modeling, and AI into clinical practice, reducing costs and increasing access to advanced techniques across various healthcare settings. The present article expands on this work by presenting a reproducible technical workflow, enhanced with AI-assisted Blender scripts, and demonstrating its application in a clinical case involving PMEG. This streamlined approach may help shorten preoperative planning time and make FEVAR more accessible in non-tertiary centers. Furthermore, the study aims to share an AI-assisted Blender script to facilitate the adoption of this workflow in tertiary hospitals and promote broader implementation of patient-specific planning techniques.

## Case presentation

**Figure 1:**
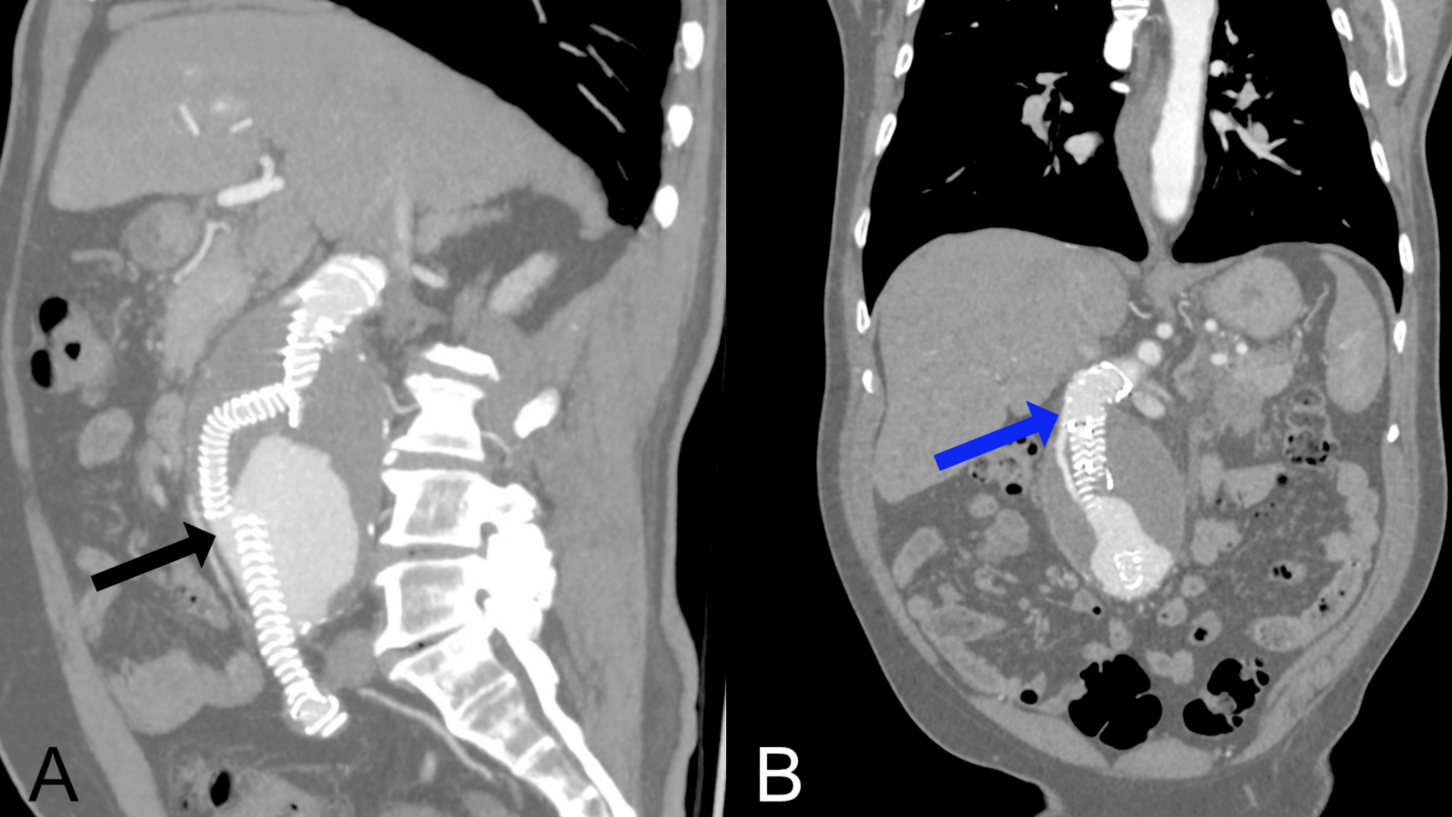
Postoperative CT angiography at presentation showing type 1a and type 3a endoleaks (A) Multiplanar reconstruction in a predominantly sagittal plane with maximum intensity projection (MIP) showing a type 3A endoleak (black arrow) caused by disconnection between the limb extension and the branch of the Anaconda bifurcated endograft. (B) Multiplanar reconstruction in a mainly coronal plane with MIP demonstrating a type 1A endoleak (blue arrow) originating from the proximal seal zone and extending into the aneurysmal sac.

A 78-year-old man with a 50-pack-year smoking history was admitted to the emergency department with abdominal pain and recurrent vomiting. Laboratory tests confirmed acute edematous pancreatitis. An abdominal CTA showed pancreatitis and an incidental 70 mm infrarenal AAA. After recovering from pancreatitis, the patient was scheduled for EVAR. The initial EVAR, performed at another facility using an Anaconda ALP 28 endograft (Terumo Aortic, Inchinnan, United Kingdom) via bilateral femoral access, was technically successful. However, follow-up CTA revealed a type Ia endoleak and a type IIIa endoleak at the left iliac extension (Figure 1), leading to a referral for reintervention at our institution.

Preoperative planning began with a high-resolution CTA of the abdominal aorta, performed according to a standard EVAR protocol with a slice thickness of 1 mm or less. DICOM files were imported into Horos. Using the curved multiplanar reconstruction (MPR) tool in stretched mode, a centerline was created by sequentially placing points along the vessel lumen. This method preserves isometry and allows accurate measurement of length and angles, which are essential for fenestrated endograft planning. Vessel measurements were taken using the arc-length method to obtain precise distances along the true vascular curvature, thereby ensuring correct localization of visceral branch origins.

**Figure 2:**
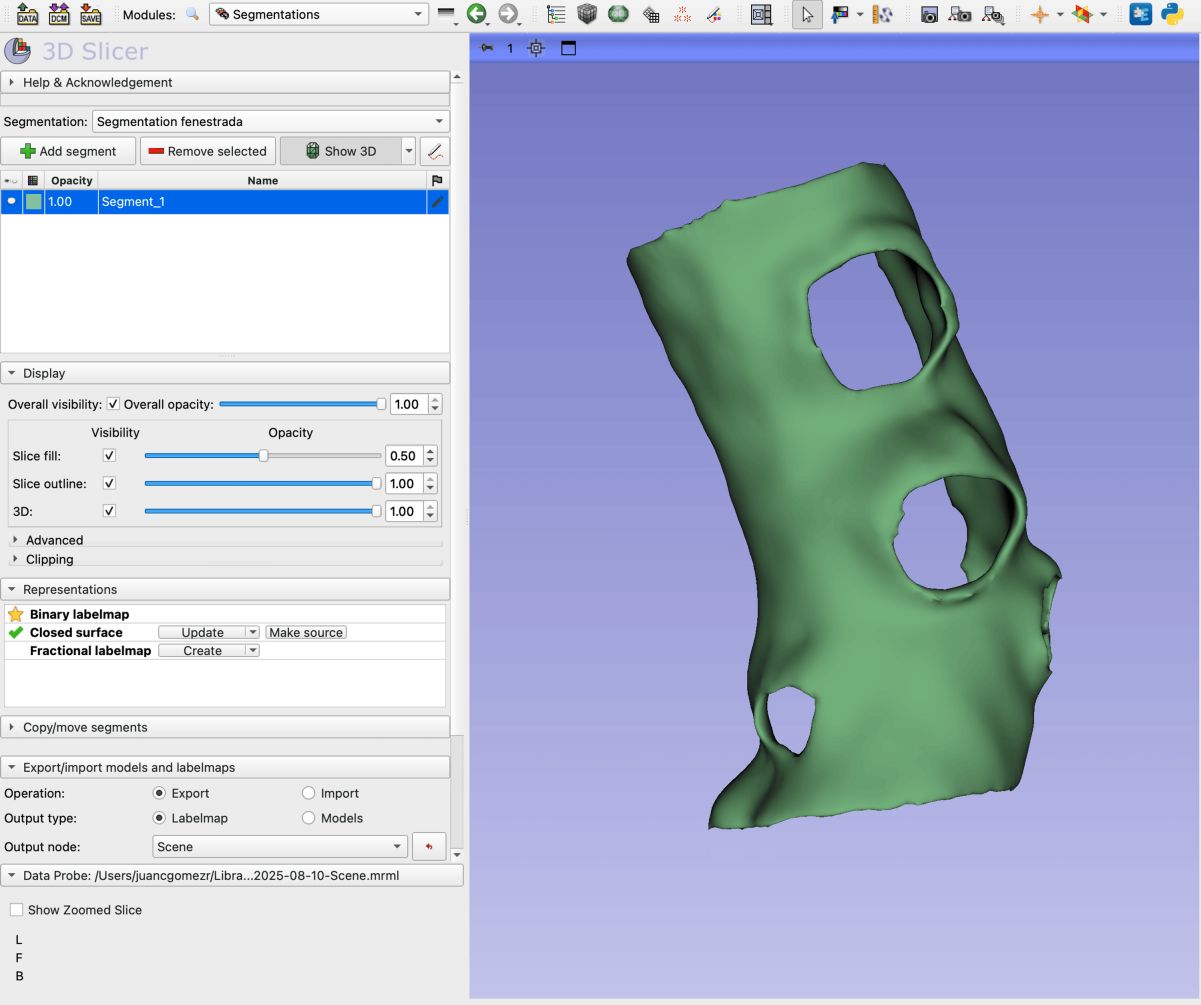
Screenshot of the 3D Slicer Segmentation module showing the fenestrated aortic model Screenshot from 3D Slicer displaying the Segmentation module interface. The left panel shows the control menus, including Display, Representations, and Copy/Import segment options. On the right, the 3D view presents the hollowed aortic model generated from the Computed Tomography Angiography (CTA) Digital Imaging and Communications in Medicine (DICOM) dataset using the Grow from Seeds segmentation and further processed with the Hollow tool. A precision cut was made with the Scissors tool at the level of the visceral ostia, creating the open-ended appearance of the celiac trunk, superior mesenteric artery, and renal arteries.

CTA datasets were then imported into 3D Slicer for three-dimensional reconstruction. The abdominal aorta and visceral branches were segmented using the Grow from Seeds algorithm, which enables quick and reproducible segmentation with minimal manual correction [17,18]. The model was hollowed to a wall thickness of 1 mm and sectioned longitudinally to create an anatomical replica that preserved the visceral ostia (Figure 2).

The segmentation was exported as an STL file for further processing.

**Figure 3:**
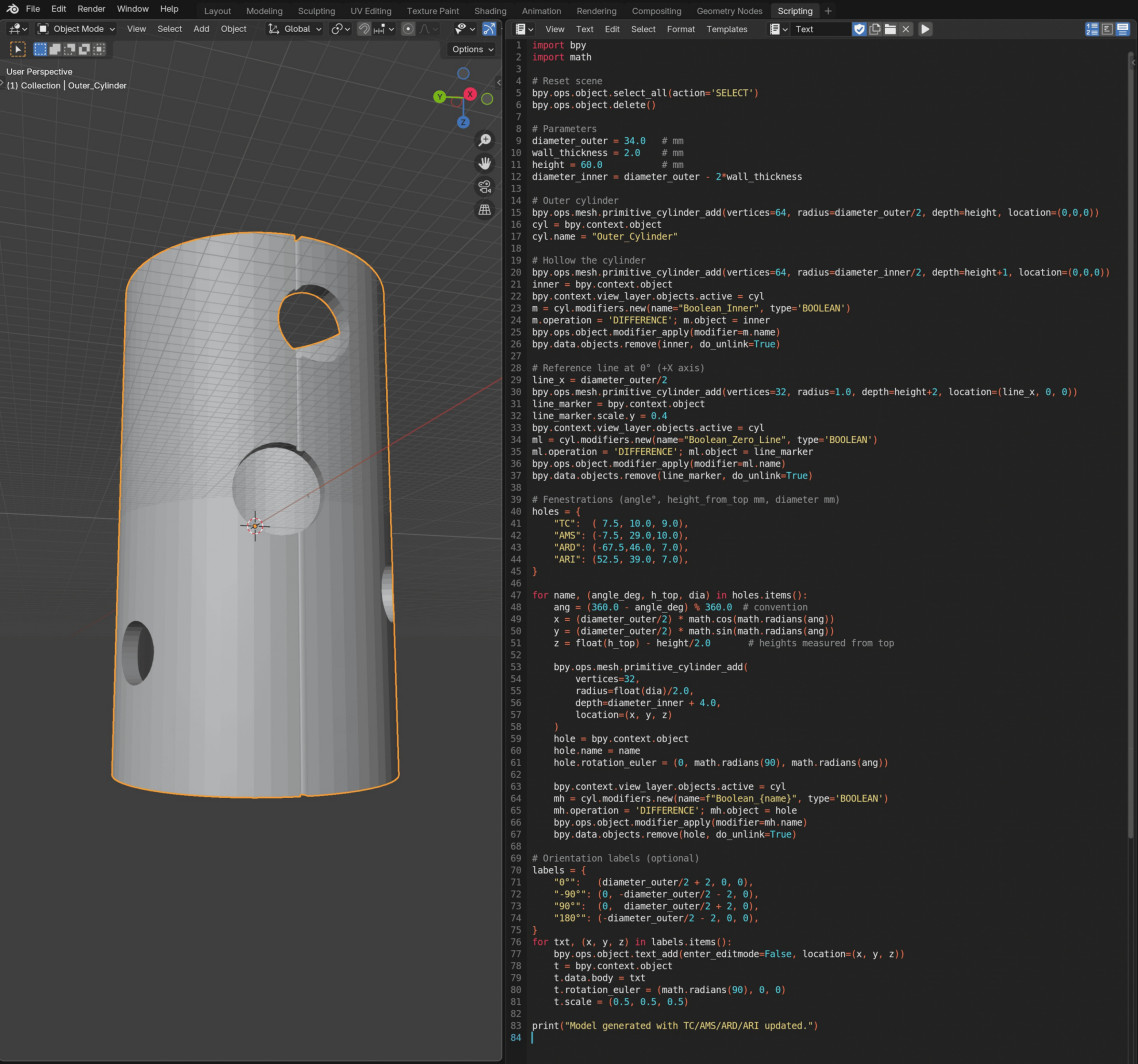
Python Script and 3D model of the fenestrated cylindrical template in Blender Screenshot from Blender showing the 3D view (left) and the Python scripting interface (right). The script automatically generates a fenestrated cylindrical template representing the aortic segment, with circular openings corresponding to the celiac trunk, superior mesenteric artery, and both renal arteries. The parameters define the outer and inner diameters, wall thickness, and fenestration angles relative to a 0° reference line. The resulting model replicates the surgical fenestration planning process used for patient-specific endograft modification.

Vessel measurements from Horos were compiled into a standardized spreadsheet and processed through an AI-based natural language model (ChatGPT, OpenAI, San Francisco, CA, USA), which generated a custom Python (Python Software Foundation, Delaware, USA) script for Blender (Appendix). The script created a parametric cylindrical aortic model scaled 1:1 to the patient’s anatomy, with fenestrations positioned at the recorded exact distances, diameters, and angles (Figure 3).

The superior mesenteric artery fenestration was set at 0° (midline anterior), with others rotated proportionally along the Z-axis. Boolean Difference operations carved out the fenestrations, and the final STL was exported at 0.1 mm resolution. The Python script used to create the model is included as supplementary material (Appendix) for reproducibility.

Both STL files (anatomical and cylindrical) were prepared in Creality Slicer (Creality 3D, Shenzhen, China) and printed on a Creality Ender 3 V2 fused-deposition modeling (FDM) printer with polylactic acid (PLA) filament. The anatomical model required supports, with an estimated print time of one to 1.5 hours, while the cylindrical fenestrated model needed minimal supports and printed in 0.5 to one hour. Both models were sterilized using low-temperature hydrogen peroxide plasma sterilization before surgical use.

**Figure 4:**
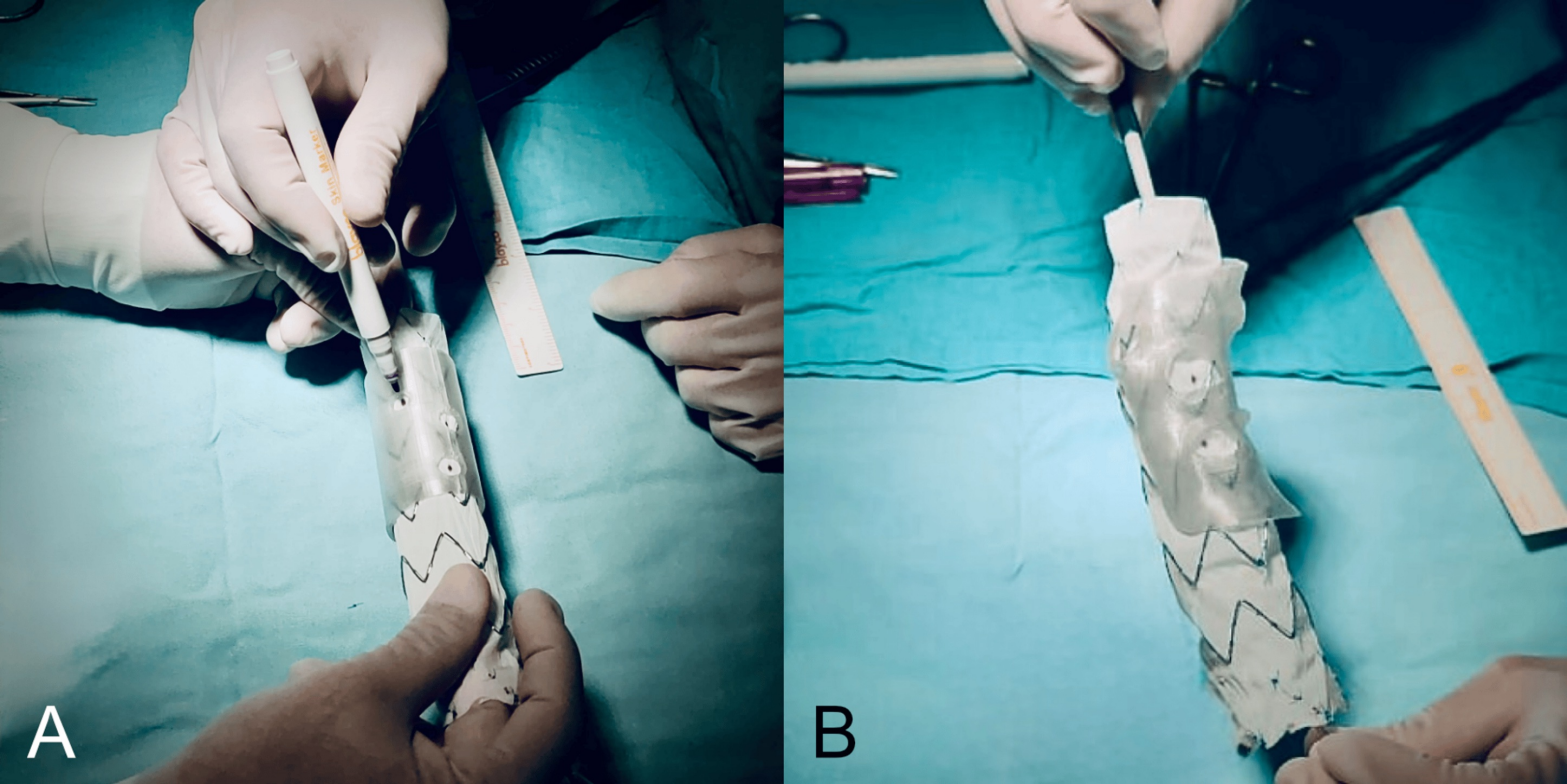
AI-assisted 3D planning and surgical marking of a patient-specific fenestrated endograft (A) Marking of fenestrations on the endograft fabric using the 3D-printed cylindrical stencil, generated from centerline-based arc-length measurements obtained from the preoperative CTA. The stencil was produced through a Python script created with the assistance of artificial intelligence (ChatGPT) and executed in Blender, resulting in a patient-specific guide printed at true scale for intraoperative use. (B) Validation of alignment between the anatomical 3D-printed model reconstructed in 3D Slicer from DICOM data and the fenestrated cylindrical stencil, confirming the accuracy and reproducibility of the AI-assisted workflow. The marked sites on the graft fabric precisely correspond to the visceral ostia, ensuring accurate positioning for surgeon-modified endograft (PMEG) fabrication.

Following preoperative planning, all patients provided informed consent for modifications to the endografts as part of the surgical planning process. The 3D-printed devices were used solely as external measurement templates-similar to a surgical ruler or stencil-to ensure accuracy during intraoperative preparation and were not in contact with the patient at any time. A PMEG was then prepared under general anesthesia and invasive monitoring using a Cook ZTA-P-30-155 thoracic endograft (Cook Medical, Bloomington, IN, USA). The sterile 3D-printed cylindrical stencil was positioned over the surface of the endograft fabric, and each planned fenestration was precisely marked with a sterile surgical marker through the corresponding openings of the stencil. These markings delineated the exact locations for the visceral vessels, ensuring correct alignment with the preoperative plan (Figure 4).

**Figure 5:**
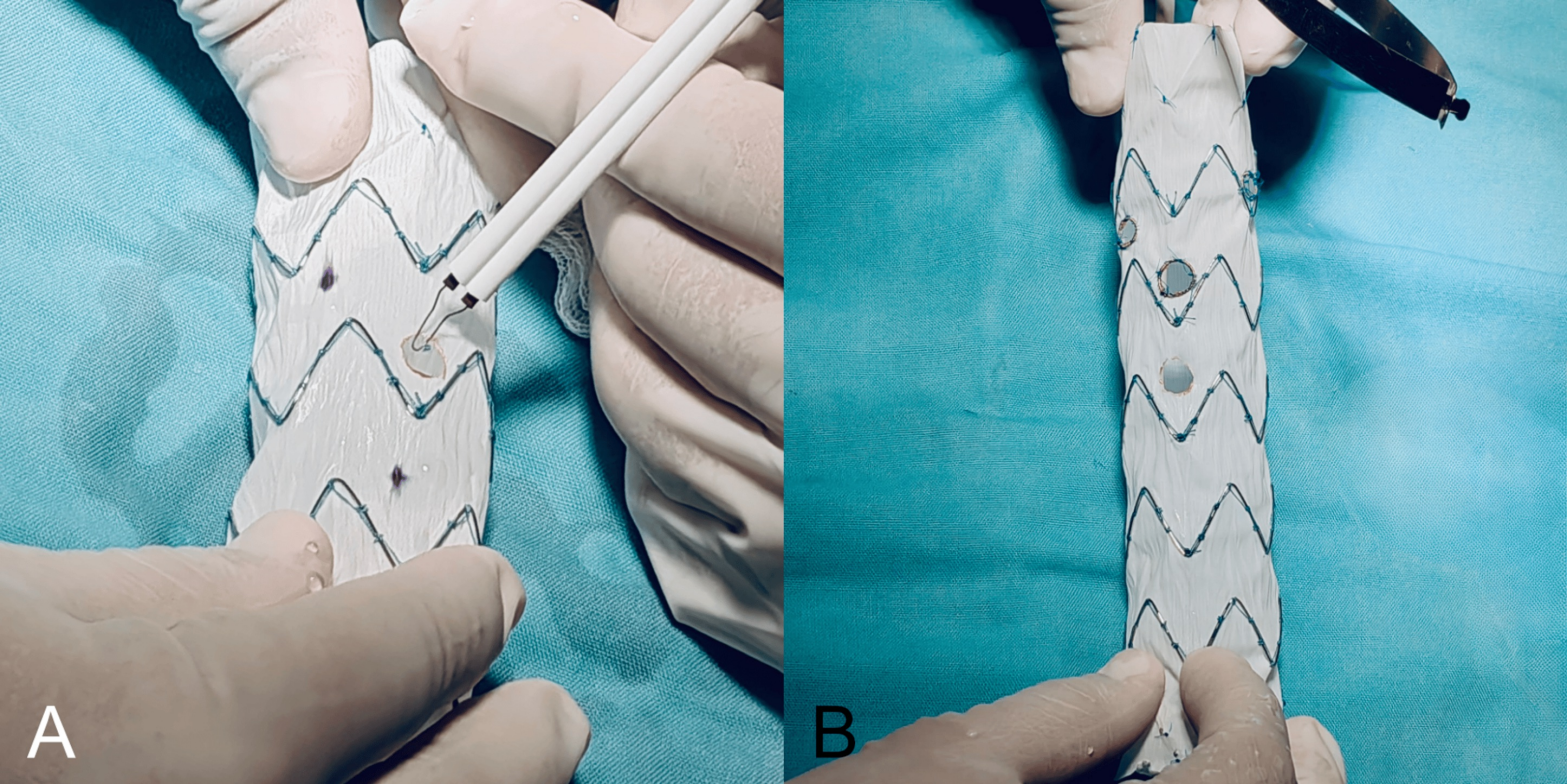
Creation and reinforcement of fenestrations on the surgeon-modified endograft (A) Fenestrations were created on the endograft fabric using electrocautery, following the previously marked positions from the AI-assisted 3D-printed stencil. (B) Each fenestration was reinforced with Prolene sutures and fitted with radiopaque markers secured with Amplatz Goose Neck snares, completing the preparation of the surgeon-modified endograft (PMEG).

Once the sites were identified, the fenestrations were created on the endograft fabric using electrocautery (FIAB, Florence, Italy) and reinforced with Prolene sutures (Ethicon, Somerville, NJ, USA). Radiopaque markers were then secured around each fenestration using Amplatz Goose Neck snares (Medtronic, Minneapolis, MN, USA) (Figure 5).

The modified graft was subsequently reloaded into its original delivery system for implantation.

Surgical access was obtained through a left subclavian cutdown and bilateral percutaneous femoral punctures. Under fluoroscopic guidance with image fusion, the fenestrations were oriented and aligned with the corresponding visceral arteries. Selective cannulation of the celiac trunk, superior mesenteric artery, and both renal arteries was achieved. Covered stents were then deployed: Begraft Peripheral (Bentley InnoMed, Hechingen, Germany) 7 × 37 mm in the left renal artery; Begraft 6 × 28 mm and 6 × 22 mm in the right renal artery; iCover (iVascular, Barcelona, Spain) 9 × 37 mm in the superior mesenteric artery; and iCover 10 × 37 mm in the celiac trunk.

To repair the type IIIa endoleak identified at the iliac bifurcation, an Anaconda iliac extension AFL1213X150 was deployed, achieving complete correction of the disconnection responsible for the endoleak. No additional reinforcement was necessary. Final angiography confirmed complete exclusion of the aneurysm, restoration of aorto-iliac continuity, patency of all visceral branches, and full correction of the earlier type IIIa endoleak, with no residual leaks.

**Figure 6:**
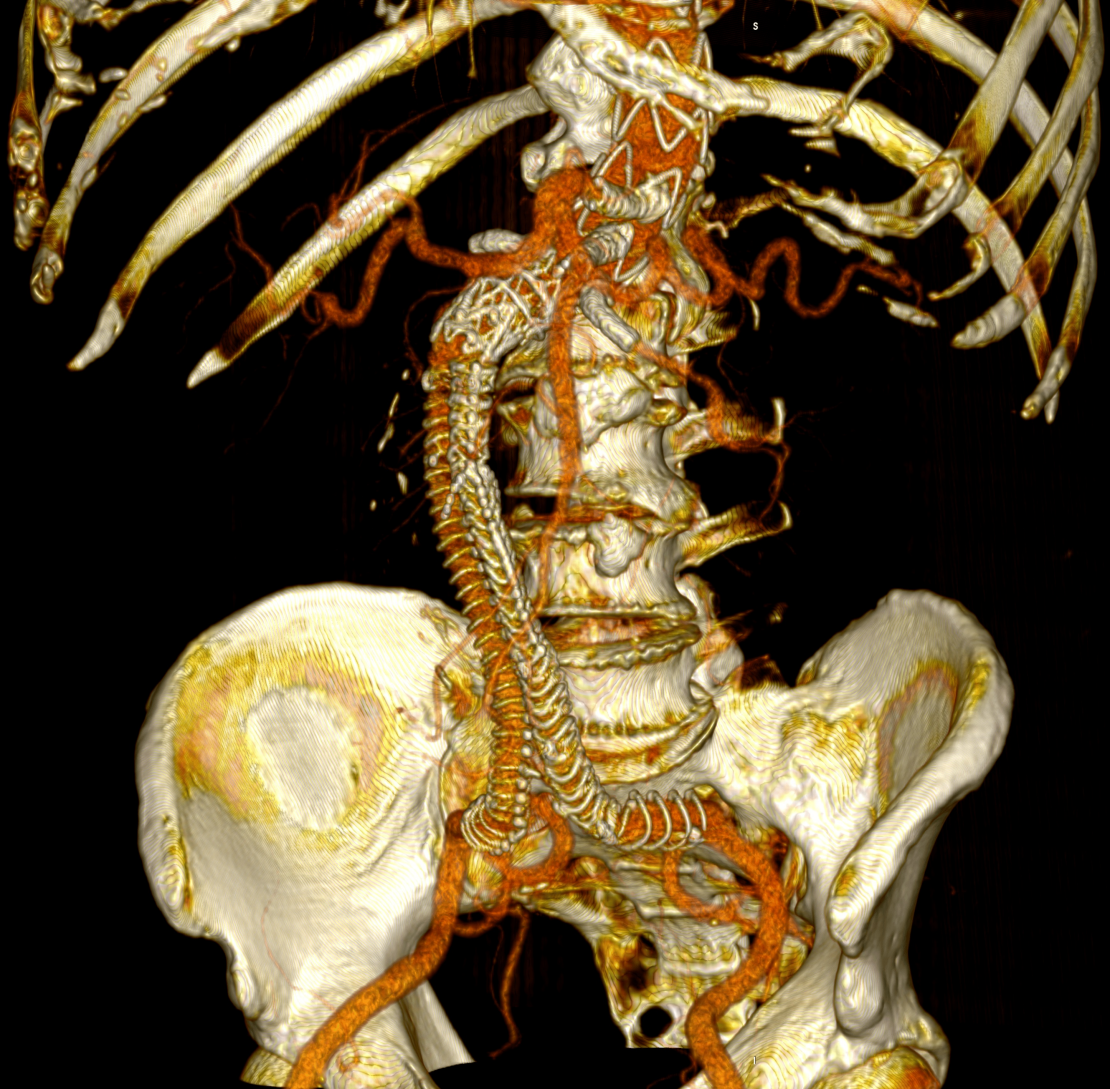
Postoperative 3D volume rendering of the fenestrated endograft reconstruction Three-dimensional volume-rendered reconstruction obtained in Horos using the standard soft-tissue preset. The image displays the postoperative appearance of the fenestrated endograft and visceral stents, extending from the descending thoracic aorta to the iliac bifurcation, acquired during the follow-up CTA one month after the procedure. The reconstruction shows patent visceral branches with well-perfused stents, absence of endoleak, and successful restoration of the previously disconnected left limb with adequate iliac perfusion.

All femoral accesses were closed with Proglide devices (Abbott, Santa Clara, CA, USA), and the subclavian arteriotomy was repaired with direct suture and vascular sealant. The patient was extubated in the angiography suite and transferred to the intensive care unit for hemodynamic monitoring, and later moved to the general ward. The postoperative course was uneventful, with no ischemic or hemorrhagic complications, adequate pain control, and good oral tolerance. The patient was discharged three days post-procedure in stable condition. A follow-up CTA obtained one month later confirmed complete exclusion of the aneurysm, absence of endoleaks, and patent visceral branches with preserved perfusion (Figure 6).

## Discussion

FEVAR/BEVAR has become an established strategy for treating juxtarenal and pararenal AAAs, offering durable exclusion while maintaining visceral perfusion. Large international series have reported high technical success rates, acceptable perioperative morbidity, and sustained branch vessel patency [1-3]. However, widespread adoption remains limited by the high cost and manufacturing delays of custom-made fenestrated devices, which often make them unsuitable for urgent or resource-limited settings.

In this context, PMEGs have emerged as a practical alternative, especially for urgent or anatomically complex cases. Reports from various centers confirm the feasibility of PMEGs with low perioperative mortality and promising mid-term results [20]. Still, their success depends heavily on thorough preoperative planning, precise fenestration alignment, and intraoperative accuracy. The present case shows how digital tools can improve this workflow by reducing variability and enhancing reproducibility.

3D printing has grown increasingly important in aortic repair for training, simulation, and procedural planning [6,7]. In fenestrated EVAR, printed templates guide fenestration marking with high accuracy, improving alignment and reducing operative time. Our workflow produced two complementary models: a patient-specific anatomical reconstruction generated in 3D Slicer, which provides an intuitive understanding of branch vessel relationships, and a cylindrical, fenestrated model created automatically via AI-assisted Blender scripting, serving as a quick intraoperative stencil for PMEG modification. This dual-model approach, seldom reported in literature, adds educational and practical value to the planning process.

Commercial vascular planning platforms are expensive and often unavailable in low- and middle-income regions. Open-source options such as Horos, 3D Slicer, and Blender democratize access to advanced imaging and modeling tools. In our workflow, Horos enabled precise centerline-based measurements, 3D Slicer produced accurate anatomical segmentations, and Blender enabled automated scripting for fenestration placement. Similar combinations have been reported in prior Latin American studies [17-19], including our work on cost-effective 3D printing for aneurysm planning and template creation. This study builds on that foundation by incorporating AI automation into the process. To promote reproducibility and encourage adoption, the complete Blender Python script used to generate the cylindrical fenestrated template is provided as supplementary material (Appendix). Sharing this script publicly not only demonstrates the potential for surgeon-led digital innovation but also highlights the clinical relevance of this workflow, which can streamline preoperative preparation and potentially expand access to FEVAR in community hospitals by reducing dependence on proprietary planning platforms.

AI is increasingly used in vascular surgery, from automated image segmentation to predictive analytics [14-16]. In this case, AI was employed not for image analysis but for workflow automation, transforming a structured spreadsheet of vessel measurements into a Python script that generated a parametric Blender model within seconds. This innovative use of AI demonstrates how automation can connect clinical data with tangible intraoperative tools, accelerating innovation without requiring advanced programming skills.

Using a Creality Ender 3 V2 printer with PLA filament further demonstrates that effective intraoperative templates can be produced with affordable consumer hardware. The anatomical model took about 1.5 hours to print, while the cylindrical fenestrated model took 0.5 to 1 hour, making it feasible even for urgent procedures. With a material cost under USD 5 per model, this method is much cheaper than commercial options, supporting the global push toward cost-effective, scalable innovation in vascular surgery.

Several limitations should be recognized. First, fenestration alignment accuracy still depends on careful CTA-based measurements, and errors can be transferred to the printed model. Second, PLA models cannot tolerate high-temperature sterilization and are limited to low-temperature plasma or ethylene oxide sterilization methods. Third, although this workflow is quick and reproducible, it requires familiarity with multiple open-source platforms, which may pose a learning curve for surgical teams. Future advances could include fully automated pipelines from CTA acquisition to STL export, further reducing preparation times. AI-based segmentation and vessel detection tools are approaching clinical maturity and could be seamlessly integrated into this process. Validation studies comparing the accuracy of AI-assisted cylindrical templates with traditional manual planning are also needed.

## Conclusions

This case illustrates that a fully open-source, AI-assisted digital workflow can enable accurate and reproducible planning for physician-modified endografts at a fraction of the cost of commercial systems. By integrating Horos for centerline extraction, 3D Slicer for anatomical segmentation, Blender for AI-driven parametric modeling, and desktop 3D printing for template fabrication, this process converts digital data into precise intraoperative guides.

The dual-model approach - combining a patient-specific anatomical replica with a cylindrical fenestrated stencil - enhances spatial understanding, improves intraoperative precision, and supports safer, faster modification of endografts. Beyond its technical feasibility, this method highlights the potential of surgeon-led digital innovation to expand access to advanced endovascular repair in centers without commercial planning platforms or custom-made devices.

Ultimately, this workflow offers a scalable, low-cost, and reproducible solution that bridges engineering and surgical practice, advancing personalized aortic repair through open-source and AI-enabled design. Future studies should focus on validating this workflow across larger patient cohorts and diverse clinical settings, and on refining automation tools to further streamline preoperative planning and expand accessibility.

## Appendices

Custom Python script for Blender

# Blender Python Script - Fenestrated Cylinder

import bpy

import math

# Reset scene

bpy.ops.object.select_all(action='SELECT')

bpy.ops.object.delete()

# Parameters

diameter_outer = 34.0   # mm

wall_thickness = 2.0    # mm

height = 60.0           # mm

diameter_inner = diameter_outer - 2*wall_thickness

# Outer cylinder

bpy.ops.mesh.primitive_cylinder_add(vertices=64, radius=diameter_outer/2, depth=height, location=(0,0,0))

cyl = bpy.context.object

cyl.name = "Outer_Cylinder"

# Hollow the cylinder

bpy.ops.mesh.primitive_cylinder_add(vertices=64, radius=diameter_inner/2, depth=height+1, location=(0,0,0))

inner = bpy.context.object

bpy.context.view_layer.objects.active = cyl

m = cyl.modifiers.new(name="Boolean_Inner", type='BOOLEAN')

m.operation = 'DIFFERENCE'; m.object = inner

bpy.ops.object.modifier_apply(modifier=m.name)

bpy.data.objects.remove(inner, do_unlink=True)

# Reference line at 0° (+X axis)

line_x = diameter_outer/2

bpy.ops.mesh.primitive_cylinder_add(vertices=32, radius=1.0, depth=height+2, location=(line_x, 0, 0))

line_marker = bpy.context.object

line_marker.scale.y = 0.4

bpy.context.view_layer.objects.active = cyl

ml = cyl.modifiers.new(name="Boolean_Zero_Line", type='BOOLEAN')

ml.operation = 'DIFFERENCE'; ml.object = line_marker

bpy.ops.object.modifier_apply(modifier=ml.name)

bpy.data.objects.remove(line_marker, do_unlink=True)

# Fenestrations (angle°, height_from_top mm, diameter mm)

holes = {

    "TC":  ( 7.5, 10.0, 9.0),

    "AMS": (-7.5, 29.0,10.0),

    "ARD": (-67.5,46.0, 7.0),

    "ARI": (52.5, 39.0, 7.0),

}

for name, (angle_deg, h_top, dia) in holes.items():

    ang = (360.0 - angle_deg) % 360.0  # convention

    x = (diameter_outer/2) * math.cos(math.radians(ang))

    y = (diameter_outer/2) * math.sin(math.radians(ang))

    z = float(h_top) - height/2.0       # heights measured from top

    bpy.ops.mesh.primitive_cylinder_add(

        vertices=32,

        radius=float(dia)/2.0,

        depth=diameter_inner + 4.0,

        location=(x, y, z)

    )

    hole = bpy.context.object

    hole.name = name

    hole.rotation_euler = (0, math.radians(90), math.radians(ang))

    bpy.context.view_layer.objects.active = cyl

    mh = cyl.modifiers.new(name=f"Boolean_{name}", type='BOOLEAN')

    mh.operation = 'DIFFERENCE'; mh.object = hole

    bpy.ops.object.modifier_apply(modifier=mh.name)

    bpy.data.objects.remove(hole, do_unlink=True)

# Orientation labels (optional)

labels = {

    "0°":   (diameter_outer/2 + 2, 0, 0),

    "-90°": (0, -diameter_outer/2 - 2, 0),

    "90°":  (0,  diameter_outer/2 + 2, 0),

    "180°": (-diameter_outer/2 - 2, 0, 0),

}

for txt, (x, y, z) in labels.items():

    bpy.ops.object.text_add(enter_editmode=False, location=(x, y, z))

    t = bpy.context.object

    t.data.body = txt

    t.rotation_euler = (math.radians(90), 0, 0)

    t.scale = (0.5, 0.5, 0.5)

print("Model generated with TC/AMS/ARD/ARI updated.")
